# Calling situated: a survey among medical students supplemented by a qualitative study and a comparison with a surveyed sample of physicians

**DOI:** 10.1186/s12909-022-03642-x

**Published:** 2022-08-15

**Authors:** S. Bonvin, F. Stiefel, M. Gholam, C. Bourquin

**Affiliations:** 1 Psychiatric Liaison Service, Lausanne University Hospital and University of Lausanne, Lausanne, Switzerland; 2 Center of Epidemiological Psychiatry and Psychopathology (CEPP), Lausanne University Hospital and University of Lausanne, Lausanne, Switzerland

**Keywords:** Calling, Medical students, Study choice, Choice consistency, Undergraduate medical education

## Abstract

**Background:**

Calling within the medical context receives growing academic attention and empirical research has started to demonstrate its beneficial effects. The purpose of this study is to investigate what motivates students to enter medical school and what role calling may play (i), to evaluate if calling influences the way in which they experience their studies (ii), and to compare medical students’ experience of calling with those of physicians.

**Methods:**

A questionnaire survey was distributed among medical students (*N* = 1048; response rate above 60%) of the University of Lausanne in Switzerland. It was supplemented by a group discussion between bachelor medical students (*N* = 8) and senior physicians (*N* = 4), focusing on different facets of calling. An existing data set of a survey among physicians, addressing calling with the same questionnaire, was used to compare students’ and physicians’ attitudes towards calling. Survey data were analyzed with the habitual statistical procedures for categorical and continuous variables. The group discussion was analyzed with thematic analysis.

**Results:**

The survey showed that experiencing calling is a motivational factor for study choice and influences positively choice consistency. Students experiencing calling differed from those who did not: they attributed different definitions to calling, indicated more often prosocial motivational factors for entering medical school and perceived the learning context as less burdensome. The analysis of the group discussion revealed that the concept of calling has a fluid definition. It was conceived as having the characteristics of a double-edged sword and as originating from within or outside or from a dialectic interplay between the inner and outer world. Finally, calling is experienced less often by physicians than by medical students, with a decreasing prevalence as the immersion in the clinical years of the study of medicine progresses.

**Conclusions:**

Calling plays an important role in study choice and consistency of medical students. Given its relevance for medical students and its ramifications with the learning context, calling should become a topic of the reflexive parts of the medical curriculum. We critically discuss the role played by calling for medical students and provide some perspectives on how calling could be integrated in the reflection and teaching on physicianhood.

## Background

*“This year matriculated students are particularly numerous and this preoccupies me. The auditorium was designed for 500 persons and they are now 730 to attend”* [1]. This statement of the Director of the Medical School of Lausanne University (Switzerland) has been published in local newspaper. It highlights the steadily increasing number of high school graduates who choose to enter medical school, even though medical studies are reputed to be arduous due to a high first-year failure rate and a heavy workload.

What motivates these young people to start such challenging studies and what allows them to resist the pressures of the highly selective first year and the hardships of the subsequent years?

Most empirical studies investigating medical career choice focus on determinants of undergraduate academic performance and success or on motives for postgraduate specialization [2]. The literature on *choice of studying medicine* is rather scarce, as shown by a systematic review of the literature [3]. The authors reported that choice varies according to national economic situations and link this finding to Maslow’s pyramid of needs [3]. This pyramid consists of a first level of basic needs, such as eating or sleeping (i), on which follows a need for security, stability and protection (ii), being part of a community or to have a family (iii), and a fourth level with two segments: respect and prestige (a) and trust in oneself and personal development (b). In high-income countries [4–9], the principal motivations for studying medicine are an interest for science/medicine, social and professional independence, and the anticipated variety of the work as physician. In countries with moderate incomes, social factors, such as influence from the family and expectations with regard to the social status, as well as scientific interest determine study choice [3]. In lower-income countries [10, 11], humanitarian factors, such as helping others and society prevail.

The stakes related to these personal and social motivations and the important pressure encountered in the studies may thus explain the high prevalence of burnout, depression and anxiety in medical students [12–14], as confirmed by a systematic review [14]. Academic pressure, heavy workload, financial concerns, sleep deprivation and the “hidden curriculum”^1^ were identified as specific causes of these mental health problems [14]. Moreover, studies reported a constant decrease of interest for the medical profession over the study years [17], a drop of idealism and a raise of cynicism [18, 19]. After graduation, an important minority of physicians leave medicine: in Switzerland, 5–10% of medical doctors quit their job [20, 21].

Given these observations, the question raises if factors exist, which initiate and maintain the motivation to study and to become a physician. Calling, historically associated with the medical profession [22, 23], might be such a factor. While calling appears to be a somehow outdated concept [22], the interest in calling has raised over the last years and calling has become a topic of scientific investigation. Calling was for example identified as decreasing physicians’ burnout [13, 24, 25]. Moreover, in students (not only medical students) who declare experiencing a sense of calling, positive associations have been found with well-being, autonomy, satisfaction with studies, work and life, the capacity to adapt and confidence in the study and career choice [26, 27]. Surveys revealed that the notion of calling is still popular among medical students [2, 28]. For example, a study among medical students showed that only 10% of them thought that it was not appropriate to link medicine and calling [29]. The concept of calling in medicine and among medical students thus merits interest and further investigation.

As outlined in a previous article reporting on a study among physicians, the definition of calling largely depends on the authors and tends to change over time [30, 31]. The tripartite definition by Dik and Duffy has served as a basis for many studies – including this one – and conceptualization of calling [32]. Dik and Duffy described calling as ‘‘(1) a transcendent summons, experienced as originating beyond the self, to (2) approach a particular life role in a manner oriented toward demonstrating or deriving a sense of purpose or meaningfulness and (3) that holds other-oriented values and goals as primary sources of motivation’’ [27, 31, 32].

It is worth noting that in the context of the first wave of the Covid-19 pandemic, calling reemerged more frequently in the discourses circulating within medicine, the society and the media and was used when referring to clinicians who disregarded their usual working hours or risked their life to save those of others [33]. As stated by a popular local news journal: *“Mobilized to face the virus, clinicians see their already charged working hours increase and fear exhaustion […]. Often pushed by calling, the healthcare personnel shows solidarity ….”* [34].

The aim of this study was to investigate what motivates to enter medical school and what role calling plays with regard to study choice and study consistency. A secondary objective was to investigate if medical students with and without calling have a similar or different experience of the learning context. Finally, given the fact that we have recently surveyed physicians with regard to calling, we aimed to examine whether there are differences between medical students and physicians with respect to the experience of calling.

## Methods

The study was designed as a quantitative questionnaire survey among medical students, supplemented by a qualitative investigation based on a group discussion with both medical students (*N* = 8) and senior physicians (*N* = 4) and focused on the definition, conceptualization and relevance of calling. Moreover, the results of the quantitative survey were compared with those of a previous study conducted among physicians.

### Participants

Participants were Bachelor (B) and Master (M) students (study years 1 to 6) of Lausanne University Medical School (N = 1605 students in 2019).

### Data collection

#### Questionnaire survey

The survey was based on a questionnaire, which was used in a previous study investigating calling among physicians [35]. In a nutshell, the starting point of this previous study was the ambivalent reaction of interviewed physicians [36] to a statement about the sense of calling from a book by Jaddo, a young French general practitioner, which can be translated as follows: *We often hear: “To be a physician, you must have a calling. Calling? I don’t know what it is. I have never seen it. Nobody introduced me to it. And no doctor or student has ever told me that he/she has it”* [37] (translation by the authors). Details are reported in the article on physicians’ calling [35]The constructs and measurement items that were used in the developed questionnaire are also presented in this last article [35]. We slightly modified the questionnaire for the students to include items addressing the learning context of medical students, with the aim to generate a situated view of the medical student and calling.

The survey contained open- and close-ended questions with response options or a response scale. It consisted of five sections: personal information (e.g., gender, year of study); motivators for choosing medicine (e.g., scientific aspects, a certain altruism); study choice and consistency of choice (e.g., medicine as the only choice of study or not, desire to choose medicine again); definitions of calling (e.g., a predisposition, a passion, a sacrifice, etc.) and the level of agreement with the statement about calling by Jaddo; and experience of the learning context (e.g., benefits of studying medicine) (see Appendix 1). By learning context we mean the determinants and conditions, which influence students’ experiences of their studies [36]. The learning context was assessed by means of data from the literature [38, 39] and our own experience as medical professionals and medical students. The present study, like the one on physicians [35], was driven by a desire to further explore how physicians and medical students situate themselves in relation to calling, and thereby how they individually comprehend and define this concept. Therefore, we did not provide them with an articulated definition of calling from us or from the literature or use validated questionnaires link to a specific definition.

We used a paper-based version of the questionnaire for B1 to B3 and M1 and M2 students, and an online questionnaire survey (LimeSurvey®) for M3 students who are in internships in different hospitals. The survey, completed anonymously, was conducted between October and November 2019, except for B1 students who responded in March 2020 (in the second half of their academic year in order not to include respondents who leave medical school early for another career).

#### Group discussion

To supplement the survey data with qualitative data, a group discussion with B3 medical students (N = 8) and senior physicians (N = 4; two psychiatrists, a surgeon and a public health specialist) was organized with the aim to further explore the different facets of calling and deepen its definition. Physicians were selected based on their interest for pre-graduate teaching, two of them participated in the B3 optional course on physicianhood during which the discussion took place. Participants were invited to discuss specific aspects of calling, such as its definition, meanings, relevance and their use of the concept. Among the questions were the following: What is your definition of calling?; Is calling static or can it be acquired or lost in the course of one’s studies or career?; Is calling as a concept still makes sense in the 2020’s? Moreover, the following sentence from a philosophical thesis on calling in medicine was used to discuss calling as a professional quality: “*to say that a physician”has a calling” is without doubt, in the general population, a way to make of him a “good physician*” [23].

The discussion lasted two hours and took place in March 2020, with SB and CB operating as facilitators. Students and physicians, facing each other, responded to questions and statements and interacted with each other. The discussion was audio-recorded and transcribed verbatim.

The study was not based on a mixed-method approach. The research questions of the quantitative survey and of the qualitative group interview were indeed different, and the qualitative investigation mainly aimed to gain insights into the dynamics of calling, which is not possible with quantitative methods.

#### Existing data

The medical student survey data were compared with a dataset of Swiss physicians (*N* = 283, 144 females) who completed the same questionnaire (except for the section addressing the learning context) as part of a previous survey [35]. Medical specialties with the highest numbers of respondents were general internal medicine (*n* = 118), adult and child-adolescent psychiatry (*n* = 43), anesthesiology (*n* = 31) and surgery (*n* = 22). Those with fewer respondents were in an “others” category (*n* = 69). Respondents were chief residents (*n* = 96), residents (*n* = 84), senior staff members (*n* = 54) and chiefs of service (*n* = 14), and 35 respondents were physicians in private practices. Almost three-quarters of the participants worked in a university hospital. The median year of graduation from medical school was 2008 and ranged from 1974 to 2018.

### Data analysis

#### Statistical analyses

To identify associations between the presence of calling and categorical variables (i.e. gender, years in university, motivations for the profession, the study context, the process of choice (career choice and choice consistency) and the definitions attributed to calling), we used Pearson’s chi-squared test of independence. This test is valid if the expected frequencies of more than 80% cells are above five. We used Fisher’s Exact Test (FET) if validity of chi-square test was not guaranteed (frequencies expected to be below five in certain cells). Continuous variables (the number of reported motivations and the degree of agreement with the statement by Jaddo) were compared between groups by using Student’s t-test for two groups (absence or presence of calling) and by ANOVA (analysis of variance) for more than two groups (i.e., years in university and study context).

All analyses were effectuated in an R environment for statistical computing [40]. Statistical tests of hypothesis were declared significant if their corresponding p-values were lower than the pre-defined level of 0.05.

As for the study about physicians [35], our study was exploratory. We had no prior hypothesis to be verified or rejected.

#### Analysis of the group discussion

The group discussion was analyzed with thematic analysis to identify core definitions and dimensions of calling. Thematic analysis is particularly suitable to clarify how phenomena are conceptualized or understood by participants [41]. Data were categorized based on a coding frame with codes grounded in the data. Themes and their interconnections were then examined and put into perspective with the definitions and results from the survey. Since we coded and analyzed a unique discussion, we did not use a software package for qualitative data analysis.

## Results

### Questionnaire survey

#### Characteristics of the participants and response rates

Of the 1048 students who filled in the questionnaire, 709 were women and 331 men (8 participants declared themselves as “non-binary”): 366 respondents were in the B1 year, 152 in B2, 153 in B3, 112 in M1, 148 in M2 and 117 in M3 (see Table 1). Given the total number of students of Lausanne University Medical School (*N* = 1605 in 2019), the overall response rate was 65%. Response rates with regard to the study years did not differ (see Table 2); the somehow lower response rate for M3 students may be due to the way data were collected in this subgroup.

**Table 1:** Characteristics of the participants

Categorical variables	Total of respondents (*n* = 1048)
Sex	
Female [%(#)] Male [%(#)] Non-binary [%(#)]	67.65 (709) 31.58 (331) 0.76 (8)
Years in university	
B1 [%(#)]	34.92 (366)
B2 [%(#)]	14.50 (152)
B3 [%(#)]	14.60 (153)
M1 [%(#)]	10.69 (112)
M2 [%(#)] M3 [%(#)]	14.12 (148) 11.16 (117)
Calling	
With a calling [%(#)]	40.84 (428)
Without a calling [%(#)]	59.16 (620)

**Table 2:** Total of students and rate of response

Years in university	Total of respondents	Total of med students, Lausanne, 2019	Rate of response
B1	366	540	68%
B2	152	254	60%
B3	153	223	69%
M1	112	157	71%
M2	148	217	68%
M3	117	214	55%
Total	1048	1605	65%

We based our total number of students on the number from 2019, as we could not obtain the exact number of the year 2020 for administrative reasons.

The results of the survey are reported in a narrative form, frequencies and levels of statistical significance are displayed in Tables 3–6.

**Table 3:** Characteristics of the participants and calling

Categorical variables	With calling (*n* = 428)	Without calling (*n* = 620)	Test statistic (d.f.^2^)	*p* value^1^
Female [%(#)] Male [%(#)] Non-binary [%(#)]	69.39 (297) 29.91 (128) 0.7 (3)	66.45 (412) 32.74 (203) 0.81 (5)	χ2(1) = 1.01	NS
Years in university				
B1 [%(#)]	46.73 (200)	26.77 (166)		
B2 [%(#)]	12.62 (54)	15.81 (98)		
B3 [%(#)]	17.06 (73)	12.9 (80)		
M1 [%(#)]	8.41 (36)	12.26 (76)		
M2 [%(#)]	10.05 (43)	16.94 (105)	χ2(5) = 69.17	< 0.0001****
M3 [%(#)]	5.14 (22)	15.32 (95)		

^*1*^*NS* no significance

^*2*^*d.f* degrees of freedom

For the comparison between students and physicians, results are not shown in a table, since two different samples cannot really be compared. However, trends with regard to similarities and differences are also reported in a narrative form.

#### Calling, study choice and study consistency

Among the different motivators for study choice, about 40% of the students indicated calling; this percentage did not differ between men and women (see Table 3). However, what differed significantly was that calling dropped over the study years: while calling was reported by slightly more than half of B1 students, in subsequent years its prevalence decreased (dropping to less than 30% in M2 students (43 students with calling of a total of 148 M2 students)) (see Table 3).

When a sense of calling was experienced, students more often indicated being motivated by *a certain altruism, an interest in the medical world* and *the sick person*, *a certain idealism* or a *life event*. The more external motivators, such as *family influences*, *social recognition* and *guarantee of a certain income*, as well as an intrinsic *need to be useful/to act*, did not differ between those who experienced calling and those who did not. Participants without calling significantly more often indicated *scientific aspects of the medicine*, *without a specific motivation* or a *default choice* as having motivated their choice (see Table 4).

**Table 4:** Study choice, choice consistency and calling (since some participants did not answer this question, the total number counts 1041)

Categorical variables	Total (*n* = 1048)	With a calling (*n* = 428)	Without a calling (*n* = 620)	Test statistic (d.f.^2^)	*p* value^1^
Career motivators					
Need to be useful/to act [%(#)]	69.95 (733)	71.02 (304)	69.19 (429)	χ2(1) = 0.32	NS
Scientific aspects of medicine [%(#)]	68.51 (718)	64.49 (276)	71.29 (442)	χ2(1) = 5.12	0.0236*
A certain altruism [%(#)]	61.45 (644)	67.76 (290)	57.10 (354)	χ2(1) = 12.36	0.0021 ***
Interest in the medical world [%(#)]	70.99 (744)	78.97 (338)	65.48 (406)	χ2(1) = 21.72	< 0.0001 ****
Interest for the sick person [%(#)]	41.79 (438)	48.6 (208)	37.10 (230)	χ2(1) = 15.46	< 0.0001 ****
A certain idealism [%(#)]	20.13 (211)	24.77 (106)	16.94 (105)	χ2(1) = 9.18	0.0022**
Family influences [%(#)]	17.56 (184)	18.46 (79)	16.94 (105)	χ2(1) = 0.30	NS
Social recognition [%(#)]	18.33 (192)	19.39 (83)	17.58 (109)	χ2(1) = 0.44	NS
Guarantee of a certain income [%(#)]	29.88 (313)	31.77 (136)	28.55 (177)	χ2(1) = 1.1	NS
Life event [%(#)]	20.71 (217)	25.23 (108)	17.58 (109)	χ2(1) = 9.65	0.008**
Without a specific motivation [%(#)]	12.3 (129)	2.57 (11)	19.03 (118)	χ2(1) = 62.06	< 0.0001 ****
Default choice [%(#)]	4.10 (43)	0.47 (2)	6.61 (41)	χ2(1) = 22.77	< 0.0001 ****
Career choice					
Medicine as a unique choice [%(#)]	45.89 (481)	65.42 (280)	32.41 (201)	χ2(1) = 110.58	< 0.0001 ****
Choice consistency: would you choose medicine again?					
Yes [%(#)]	77.43 (806)	86.52 (366)	71.2 (440)		
Hesitation [%(#)]	18.92 (197)	12.74 (54)	23.14 (143)		
No [%(#)]	3.65 (38)	0.71 (3)	5.66 (35)	χ2(2) = 38.78	< 0.0001 ****
No answer [%(#)]	0.67 (7)				

^*1*^*NS* no significance; .*p* < .1 = trend; *p < .05, **p < .01, ***p < .001 , ****p < .0001 degree of significance

^*2*^*d. f* degrees of freedom

About three quarters of the participating students would *choose medicine again*, and calling significantly and positively influenced this stance; this result is in coherence with calling also being positively and significantly associated with *medicine as a unique choice of career* (see Table 4).

#### Perception of the learning context

Perception of the learning context was ambivalent. On the one hand, studies seem to benefit students: steadily providing increasing *confidence in oneself* and *stress management abilities*, a feeling of *self-worth*, *social achievement* and *perspective of job stability*, and throughout the years a certain *solidarity*, as well as *mental stimulation* and a *feeling of fulfillment*. On the other hand, students reported *competition* (especially in the first year), *stress*, a *difficulty to recognize one’s limits*, a *loss of social life* (attenuated over the years), *frustration* and *exposition to difficult situations* (especially in the clinical years), as well as growing *discouragement*, *disillusionment*, lack of *motivation*, *desire to stop medicine* and a decrease for *confirmation of study choice* (see Table 5).

**Table 5:** Perception of the learning context and study years

Categorical variables	Total (*n* = 1048)	B1 (366)	B2 (152)	B3 (153)	M1 (112)	M2 (148)	M3 (117)	Test statistic (d.f.^2^)	*p* value^1^	
Benefits and harms of medical studies										
Confidence in oneself [%(#)]	26.43 (277)	17.21 (63)	27.63 (42)	30.07 (46)	31.25 (35)	31.76 (47)	37.61 (44)	χ2(5) = 27.93	< 0.0001 ****	
Source of stress [%(#)]	83.97 (880)	82.79 (303)	90.13 (137)	86.27 (132)	80.36 (90)	85.14 (126)	78.63 (92)	χ2(5) = 8.88	NS	
Better learning method [%(#)]	54.38 (570)	48.91 (179)	68.42 (104)	54.90 (84)	53.57 (60)	53.38 (79)	54.70 (64)	χ2(5) = 16.39	0.0058**	
Better recognition of one’s limits [%(#)]	44.94 (471)	43.17 (158)	42.11 (64)	49.02 (75)	42.86 (48)	47.30 (70)	47.86 (56)	χ2( (5) = 2.86	NS	
Discouragement [%(#)]	32.54 (341)	32.79 (120)	25.00 (38)	29.41 (45)	34.82 (39)	35.14 (52)	40.17 (47)	χ2(5) = 8.46	NS	
Stress management abilities [%(#)]	36.73 (385)	27.05 (99)	39.47 (60)	42.48 (65)	41.07 (46)	41.89 (62)	45.30 (53)	χ2(5) = 23.46	< 0.0001 ****	
Disillusionment [%(#)]	24.62 (258)	9.02 (33)	13.82 (21)	22.22 (34)	28.57 (32)	47.30 (70)	58.12 (68)	χ2(5) = 170.37	< 0.0001 ****	
Better recognition of one’s worth [%(#)]	30.25 (317)	25.41 (93)	25.66 (39)	32.68 (50)	40.18 (45)	34.46 (51)	33.33 (39)	χ2(5) = 12.88	0.0245*	
Confirmation of study choice [%(#)]	58.11 (609)	57.10 (209)	67.11 (102)	61.44 (94)	60.71 (68)	52.70 (78)	49.57 (58)	χ2(.(5) = 11.45	0.0431*	
Motivation [%(#)]	53.72 (563)	54.37 (199)	61.18 (93)	56.21 (86)	58.04 (65)	47.30 (70)	42.74 (50)	χ2(5) = 12.86	0.0248*	
Desire to stop studying medicine [%(#)]	20.80 (218)	16.94 (62)	22.37 (34)	20.92 (32)	16.96 (19)	23.65 (35)	30.77 (36)	χ2(5) = 12.23	0.0318*	
Perspective of job stability [%(#)]	38.45 (403)	24.86 (91)	41.45 (63)	43.14 (66)	50.89 (57)	50 (74)	44.44 (52)	χ2(5) = 47.61	< 0.0001 ****	
Too big of an investment [%(#)]	31.58 (331)	27.87 (102)	32.89 (50)	37.91 (58)	33.04 (37)	31.76 (47)	31.62 (37)	χ2(5) = 5.3	NS	
Solidarity [%(#)]	28.24 (296)	26.50 (97)	30.92 (47)	33.33 (51)	29.46 (33)	31.76 (47)	17.95 (21)	χ2(5) = 10.09	0.0727	
Frustration [%(#)]	37.12 (389)	42.08 (154)	29.61 (45)	35.29 (54)	42.86 (48)	31.76 (47)	35.04 (41)	χ2(5) = 11.53	0.0418*	
Feeling of fullfilment [%(#)]	32.54 (341)	26.78 (98)	38.16 (58)	34.64 (53)	37.50 (42)	31.08 (46)	36.75 (43)	χ2(5) = 10.23	0.069	
Perspective of social achievement [%(#)]	26.81 (281)	19.12 (70)	34.21 (52)	27.45 (42)	35.71 (40)	26.35 (39)	32.48 (38)	χ2(5) = 21.55	< 0.0001 ****	
Facing difficult situations/choices [%(#)]	53.34 (559)	44.11 (161)	49.34 (75)	54.25 (83)	56.25 (63)	66.22 (98)	67.52 (79)	χ2(5) = 33.22	< 0.0001 ****	
Lack of social life [%(#)]	35.02 (367)	39.18 (143)	43.42 (66)	38.56 (59)	29.46 (33)	21.62 (32)	29.06 (34)	χ2(5) = 23.34	< 0.0001 ****	
Competition [%(#)]	36.35 (381)	47.12 (172)	29.61 (45)	31.37 (48)	33.04 (37)	25.68 (38)	35.04 (41)	χ2(5) = 30.83	< 0.0001 ****	
Mental/intellectual stimulation [%(#)]	81.39 (853)	78.41 (287)	85.53 (130)	84.97 (130)	81.25 (91)	82.43 (122)	79.49 (93)	χ2(5) = 5.25	NS	
No benefit [%(#)]	0.76 (8)	1.37 (5)	0 (0)	0 (0)	0 (0)	0 (0)	2.56 (3)	χ2(5) = 11.12(!)	0.0491*	

^*1*^*NS*  no significance; .*p* < .1 = trend; *p < .05, **p < .01, ***p < .001 , ****p < .0001  degree of significance

^*2*^*d. f* degrees of freedom

#### Calling and perception of the learning context

For many characteristics of the learning context, perception did not differ between students experiencing calling and those who did not. However, students without calling more often indicated *disillusionment*, while students with calling more often indicated *motivation* and *self-worth*; students with calling also indicated more often *frustration*, *stress*, *competition* and a *lack of social life.* Despite these more negative feelings*,* students with calling more often *confirmed study choice* (see Table 6).

**Table 6:** Perception of the learning context and calling

Benefits of medical studies					
Confidence in oneself [%(#)]	26.43 (277)	27.80 (119)	25.48 (158)	χ2(1) = 0.59	NS
Source of stress [%(#)]	83.97 (880)	87.62 (375)	81.45 (505)	χ2(1) = 6.70	0.009***
Better learning method [%(#)]	54.38 (570)	56.77 (243)	52.74 (327)	χ2(1) = 1.50	NS
Better recognition of one’s limits [%(#)]	44.94 (471)	49.76 (213)	41.61 (258)	χ2(1) = 6.48	0.011*
Discouragement [%(#)]	32.54 (341)	30.37 (130)	34.03 (211)	χ2(1) = 1.38	NS
Stress management abilities [%(#)]	36.73 (385)	36.68 (157)	36.77 (228)	χ2(1) = 0	NS
Disillusionment [%(#)]	24.62 (258)	21.03 (90)	27.10 (168)	χ2(1) = 4.7	0.0301*
Better recognition of one’s worth [%(#)]	30.25 (317)	33.88 (145)	27.74 (172)	χ2(1) = 4.23	0.039*
Confirmation of study choice [%(#)]	58.11 (609)	66.82 (286)	52.10 (323)	χ2(1) = 21.96	< 0.0001 ****
Motivation [%(#)]	53.72 (563)	61.92 (265)	48.06 (298)	χ2(1) = 18.99	< 0.0001 ****
Desire to stop studying medicine [%(#)]	20.80 (218)	18.46 (79)	22.42 (139)	χ2(1) = 2.18	NS
Perspective of job stability [%(#)]	38.45 (403)	36.45 (156)	39.84 (247)	χ2(1) = 1.09	NS
Too big investment [%(#)]	31.58 (331)	31.78 (136)	31.45 (195)	χ2(1) = 0	NS
Solidarity [%(#)]	28.24 (296)	27.80 (119)	28.55 (177)	χ2(1) = 0.04	NS
Frustration [%(#)]	37.12 (389)	40.89 (175)	34.52 (214)	χ2(1) = 4.14	0.042*
Feeling of fullfilment [%(#)]	32.54 (341)	35.51 (152)	30.48 (189)	χ2(1) = 2.69	NS
Perspective of social achievment [%(#)]	26.81 (281)	26.64 (114)	26.94 (167)	χ2(1) = 0	NS
Facing difficult situations/choices [%(#)]	53.34 (559)	52.34 (224)	54.03 (335)	χ2(1) = 0.23	NS
Lack of social life [%(#)]	35.02 (367)	39.95 (171)	31.61 (196)	χ2(1) = 7.38	0.0066**
Competition [%(#)]	36.35 (381)	40.42 (173)	33.55 (208)	χ2(1) = 4.87	0.0272*
Mental/intellectual stimulation [%(#)]	81.39 (853)	82.24 (352)	80.81 (501)	χ2(1) = 0.36	NS
No benefit [%(#)]	0.76 (8)	0.7 (3)	0.81 (5)	FET^3^	NS

^*1*^*NS* no significance; .*p* < .1 = trend; *p < .05, **p < .01, ***p < .001 , ****p < .0001 degree of significance

^*2*^*d. f* degrees of freedom

^*3*^*NS* Fisher Exact Test

#### Definitions of calling

A significant majority of students disagreed with Jaddo’s statement that calling is non-existent in medicine, unsurprisingly especially those who experienced calling. Students experiencing calling more often defined it as *internal summons*, *passion*, *sense of purpose in life* or *being in the right place*. Students who did not indicate feeling a calling significantly more often associated calling to *feeling elected/being chosen*. The groups did not differ with regard to other definitions such as *external summons*, *predisposition* or *sacrifice* (see Table 7).

**Table 7:** Agreement with Jaddo’s statement and definitions of a calling

Categorical variables	Total (*n* = 1048)	With a calling (*n* = 428)	Without a calling (*n* = 620)	Test statistic (d.f.^2^)	*p* value^1^
Jaddo’s statement					
Level of agreement	5 (0—10)	4 (0—10)	5 (0—10)	t(917) = 10.37	< 0.0001 ****
Definition of calling					
Internal summons [%(#)]	48.95 (513)	54.20 (232)	45.32 (281)	χ2(1) = 7.644	0.0057**
Passion [%(#)]	66.12 (693)	77.10 (330)	58.55 (363)	χ2(1) = 43.39	< 0.0001 ****
Sense of purpose in life [%(#)]	43.41 (455)	55.37 (237)	35.16 (218)	χ2(1) = 42.71	< 0.0001 ****
Being in the right place [%(#)]	44.27 (464)	55.37 (237)	36.61 (227)	χ2(1) = 36.69	< 0.0001 ****
External summons [%(#)]	31.01 (325)	30.37 (130)	31.45 (195)	χ2(1) = 0.05	NS
Predisposition [%(#)]	22.71 (238)	21.96 (94)	23.23 (144)	χ2(1) = 0.12	NS
Sacrifice [%(#)]	13.64 (143)	16.35 (70)	11.77 (73)	χ2(1) = 4.32	0.0377*
Feeling elected/being chosen [%(#)]	9.55 (100)	3.97 (17)	13.39 (83)	χ2(1) = 26.45	< 0.0001 ****

^*1*^*NS* no significance; .*p* < .1 = trend; *p < .05, **p < .01, ***p < .001 , ****p < .0001  degree of significance

^*2*^*d. f*  degrees of freedom

#### Comparison of responses relevant to calling between medical students and physicians

Percentage of respondents experiencing calling dropped (again) between students and physicians, with only about a fifth (19%) of physicians experiencing calling (35) (compared to 55% in B1 students and 29% in M2 students). Physicians and students experiencing calling (or not), did not distinguish themselves with regard to the definitions they attributed to calling, except for *sense of purpose in life*, which did not anymore distinguish the two groups (calling/no calling) within the physician sample (35). With regard to career choice and consistency, calling was a stabilizer for the physicians as well as for the students (35).

#### Group interview

During the group discussion, three main dimensions of calling emerged, (i) calling from inside, (ii) calling as mediator between the inner and outer world and (iii) calling from outside. Other elements addressed were the temporal dimensions of calling and the various representations calling evokes in students and physicians.

#### Calling from inside

For students, even if they did not experience calling themselves, calling can come from inside as a *predisposition*, *a call*, *a sacrifice* and a *meaning* attributed to one’s life. Some associated calling with character traits, which predispose to the medical profession: *“For me, calling is more a lived internal experience to do exactly what one feels to be the best, typically what is coherent with our character”.* Others insisted on the non-voluntary character of calling: *“From my point of view, I always experienced calling as something involuntary; I get up one day and I want to do it, without knowing exactly why”.* Calling was also considered by a student as a sacrifice: *“There is exactly this idea of the gift of self. One has to do things that costs a lot”.*

For a female student, calling was a kind of resilience or strength, an internal wealth, which makes students capable to face the study-related difficulties and the profession of medicine: *“I would say that in the collective imaginary, the good physician needs to have a sense of calling, since he/she has to cross rather complicated stages, such as the study of medicine, *etc*., and thus it’s something like raw courage, which flows in his/her veins, without calling and courage he/she would not succeed”.*

For one of the psychiatrists, calling can be perceived as “*something inside us that give a meaning to our life, an objective, a goal*.” The other psychiatrist added that it is maybe the fact to “*love doing something without exactly and rationally knowing why*”.

#### Calling as a mediator between the inner and outer world

Calling was also viewed as connecting the inner and the outer world, referring to an experience of *being at one’s place*, *feeling at ease with what is going on*, a *dialectic interplay between the desire to do something, the capacity to do it and an environment, which allows the person to do what he/she wants or has to do*. The surgeon illustrated this idea of having competences to face or realize one’s destiny by providing an example from her studies and the profession: *“At one point, I decided to do it, I decided to fight and to pass my first and my second year (as a student). Something made that I realize I was able to accomplish something” (as a physician).*

According to a student, when calling is external to oneself, it has to meet the competences: *“I view calling in a religious way, as something exterior to oneself and at the same time related to the competence a person has consciously or unconsciously”.* Lastly, this student made explicit the encounter between the inner and outer world: “*it reassures me to know what I will do in my life, to have a path to follow, and in that sense I think that it starts from something inside and from a call too.*”

#### Calling from outside

One of the psychiatrists observed: “*I am always sensitive to the idea that there is something above us.”* In line with this idea was the following comment of a student: *“I hear something telling me that I want to do this”.* Less transcendent and more immanent, calling was also understood as social reproduction within the family or as a mission assigned by society. Going a step further, a student stated *“It might be that society wants the physician to have a calling to be sure that he does his job even outside his working place”.* Calling as an external summons was also viewed as a projection or mean by others to please, motivate or stimulate: *“Calling might be something to be projected on someone, to compliment him” (Student).*

#### Characteristics of calling: temporality and representations

According to the participants of the group discussion, calling can be discovered, modified, get lost and evolve over a lifetime or in relation to events and difficulties encountered during the studies or work as a physician. The following statement by the surgeon illustrates this fluidity of calling: *“One starts with a calling to be a physician and a form of idealism, and then the idealism has to be adapted over time when really practicing medicine. And there is the difficulty, one has a beautiful calling and a beautiful profession, which allows to save a lot of people, but then there is also the reality of the medical profession and its impact on our training, on our life, the difficult moments, *etc.”

Calling can also be discovered after medical school, as the surgeon pursued: “*Clearly, I think that I didn’t have a calling when I first enter medical school, but now that I practice medicine, I feel that I had to be there”.*

Finally, calling appeared in the group interview as a double-edged sword, as also emphasized in the literature. For some students and physicians, calling represents a *capital*, which has *to be taken care of*, a *mean to preserve from bitterness*, *a line of conduct*. However, calling was also considered as a *burden*, a *constricting condition to be fulfilled*, an *injunction* to obey to, as stated by a medical student: *“There is an unhealthy side of calling. To give everything, to be stubborn. Something that defines us, we are defined by calling, and so we become stubborn, not seeing other possibilities anymore”.* For others, calling may be a danger one has to fight to avoid giving everything and get lost: *“One has seen the harmful effects of calling, the abuses, […] the dysfunctional private life, *etc*., with people being too committed to medicine” (student).* Calling would then be an obligation with the potential to destroy the one who fails to live up to the expectations. As stated by a student: *“There is a side which is less fun: there are some students, who start medicine with a calling, and even with a great sense of calling, well they didn’t pass into the second year […].”* This other student puts forward the idea of self-betrayal: *“having a sense of calling is really ‘you can't not do it’, and if you don't do it, it's a bit like betraying* yourself.*”*

## Discussion

This study aimed to investigate what calling means to nowadays medical students, and to assess – as indicated by the literature [17–19] – whether calling has a motivational role with regard to study choice and a protective function with regard to choice consistency. The results of our study, based on the quantitative survey, confirm both assumptions and provide some clues how calling operates. We will first discuss the different results by trying to put them in a coherent perspective, and then try to make sense of the quantitative and qualitative data we obtained by this investigation.

While medical students’ response rate has not the same importance as for other surveys, and representativeness is not such an issue, since this is an exploratory study, we consider that the high response rate somehow reflect that calling is still a relevant concept for medical students; this is in line with the literature [2, 28, 29]. Indeed, a majority of students entering medical school indicated to experience calling, a prevalence which drops steadily in the following years. We are aware that a cross-sectional survey does not allow to conclude that calling gets lost over the study years, but given our findings and what we know from the literature [42], the hypothesis that prevalence of calling diminishes over the study years seems quite sound. Our study sheds some new light on the underlying dynamics of this observation, namely the learning context, which seems to play a role in this loss of calling. Medical students reported an ambivalent experience of their studies. On the one hand, studies provide them with fulfilling experiences, acquisition of new skills and positive transformations of their self. On the other hand, the learning context is experienced as stressful, discouraging and frustrating, leading to a lack of social life and motivation, disillusionment and doubts concerning the study choice and even a desire to quit medical school. The immersion into the clinical years seems not to stop this evolution, on the contrary. The fact that prevalence of calling drops even further in physicians also speak for this hypothesis [35].

The question thus raises, if calling might be a motivational and protective factor in this harsh learning environment. Calling is not only one of the many reasons to enter medical school, calling also influences other motivational factors. Students who experience calling more often reported to be driven by *a certain altruism*, an *interest for the medical universe* and *the sick*, *a certain idealism* or *a life event*. These pro-social motivations may help to put one’s own comfort in the background and keep on going. One might question why the item *need to be useful*/*to act* does not differentiate between the two student groups. However, a need to be useful is not necessarily a prosocial motor, since it might not be devoid of self-interest. In health care professionals *the need to be useful* and do something may be related to own needs, for example to actively engage in suffering, which one has endured passively during the development [43] or be due to an incapacity to contain painful situations [44]. Interest in the *scientific aspects of medicine* — as more often reported by students without calling — may certainly also be a potent motor for study consistency, but science is not always at the center of the study, with clinical years providing a preview for the students of what will attend them in their professions. Moreover, students without a calling more often reported that their study choice did not rely on *specific motivation* or was decided *by default*, which lack motivational force. Keeping this in mind, it seems coherent that students experiencing calling significantly less often felt doubts and more often confirmed their study choice, showing thus choice consistency. This is also found among physicians experiencing calling [35], which underlines that the stabilizing effect of calling persists after the studies in the “real” medical world.

In addition, calling also influenced the perception of some of the characteristics of the learning environment. Items with positive perceptions, such as *recognition of self-worth*, *recognition of one’s own limits* or *motivation*, were significantly more often, and items indicating negative perceptions, such as *disillusionment* or *a desire to quit the studies*, were significantly less often chosen by students experiencing calling. And this, despite the higher prevalence of items chosen by those who experience calling, such as *frustration*, *loss of social life* and *feelings of competition*, which may explain why calling is also positively associated with choice consistency. The higher prevalence of *frustration*, *lack of social life* and *competition* in the sample of medical students experiencing calling may be due to the desire to pro-socially engage, which can be slowed down by the environment. Perception of reality is always influenced by man’s emotional state, a phenomenon, which has been described as “the logic of affect” [45] and which contributes, if loaded with positive emotions, to endure difficult situations. Finally, it might be unrealistic not to feel a certain ambivalence towards the study of medicine given the harsh conditions and challenges.

The motivational power of calling is also perceptible in the way students defined calling. For those who experience calling, calling was more often associated with *passion*, *sense of purpose*, *internal summons* or *being in the right place*. Such words remind of Antonowsky’s sense of coherence [46, 47], which is thought to protect from challenging and even traumatic experiences. The issue of sense and meaning in life has given birth to specific psychotherapeutic interventions [48], such as logotherapy, based on the experiences of the most prominent scholar of this orientation, Victor Frankl, who himself survived the holocaust [49, 50]. Finally, calling as *external summons*, *predisposition* or *sacrifice* seem to be consensual definitions, which probably are also in the general population associated with calling.

The epistemological approach adopted in the qualitative investigation provides new perspectives on calling in medical students, especially with regard to the different and sometimes contrasting functions or effects of calling. The qualitative data thus give some flesh to the survey responses, which operate with words, which are defined differently by participants. Calling is understood as coming from inside, from outside or existing at the interface between the inner and the outer world of the one that has been called. From within, calling was associated with resilience [51, 52], which again points to a protective factor. By helping to face adversities, calling was here experienced not only as a unidirectional “inner voice”, but also as an additional force leading to a kind of “give and take” situation. The group discussion also showed that there might be a dialectic between calling, the competences and capacities of the called and the conditions of the environment. This issue also plays a role when it comes to the more problematic sides of calling. Indeed, as it appears from the literature [53–56], calling may be a double-edged sword, which can lead to such an inner drive that the person stubbornly perseveres in a hopeless situation and then become a burden. This might also be the case with regard to the life events, more prevalently linked to calling. A survey among female mental health professionals compared with women working in other professions has identified a higher prevalence of somatic and psychiatric diseases, substance abuse and other indicators of family dysfunction in women, who become psychiatrist [43]. Acting on the present does indeed not resolve problems of the past or replace mourning [43]. Another danger with regard to calling was mentioned in the group, namely the fact that calling, when not followed by achievement of the mission, may lead to feelings of guilt or to the loss of the self. This might be especially the case when calling from outside becomes an injunction. Calling from outside may be understood, according to our group discussion, as a transcendental call (supra-individual), but also as a more immanent and “materialistic”, outer call, for example an expression of a family’s desire for social reproduction [57], a consequence of projections, or an injunction of some members of the society, who wish to be granted by “good doctors”, who never fail them. Consequently, calling has been considered as a “capital” not only to tap in, but one that one has to care for and reflect on, which might be rewarding, for example by providing fulfillment. This last characteristic of calling reminds of the last stages of Erickson’s eight stages of man [58, 59]: integrity versus despair. Again, the possible protective role of calling emerges in these thoughts. The contrasting effects of calling is a finding that merits attention. While it is not surprising that subjectivity shapes how humans face and experience the world, this subjectivity often remains unnoticed when it comes to concepts, which are morally valued or ideologically promoted and are thus considered to have only positive effects. For example, physicians are well advised to keep a certain emotional distance from patients. But is this distance always necessary, does it fulfil the patients’ or the physicians’ needs, and what effect does it have on physicians’ identity formation and behavior? The same holds for calling. Calling can be experienced as a source of striving or as a burdensome injunction. It might well be that this latter experience of calling adds to its fading away not only over time but also the study years.

Definitions of calling did not differ between students and physicians, nor did the prevalence of calling differ according to gender. From what has been said above, this seems coherent. There is no reason to believe that such a deeply rooted and intimate phenomenon, closely related to pro-social motivations of man, should differ according to profession or sex. Finally, calling cannot be understood as inherited and unchangeable trait of those who have it. As such the notion that those who experiencing calling are *feeling elected* seems wrong and not shared by those who experiencing it. On the contrary, calling was understood as fluid, depending on circumstances, professional experiences, and so on, in line with the above mentioned notion of calling as being a dialectic concept, in dialogue with the students’ or physicians’ inner and outer world.

What conclusions can be drawn from these data? Calling seems to positively influence study choice and choice consistency of medical students. These are cheering findings, given a learning context, which is experienced ambivalently and which has for some students [14, 60–63] and later for some physicians [20, 21, 64] dramatic psychological consequences. Calling, identified as a protective element, seems thus to maintain motivational forces, which tend to erode over the study years [3, 30, 31]. Understandably, scholars such as Kao et al. [28] invited to look for means to strengthen calling. One could for example thinks of discussing with physicians with a calling in medical schools, presenting their experiences. However, we would rather warn from such an endeavor, and this for two main reasons. First, an utilitarian promotion of calling, a highly intimate phenomenon, which cannot be acquired as a skill could create unnecessary pressure or feelings of guilt. This caution is confirmed by a recent study [65], which demonstrated that an utilitarian intervention among medical residents, mainly based on concepts from positive psychology and aimed to improve well-being, failed its goals. And second, as also illustrated by the before mentioned study, and repeatedly evoked by scholars: focusing on the individual may somehow obfuscate or deny the harsh context of medical schools [66], and leave the student with feelings of not being understood.

As already mentioned in a prior study on medical students’ subjectivities [60], we consider that students are not only ill prepared to face their later profession, there is also a general tendency to limit the study of medicine to the acquisition of knowledge, and almost no or only minimal efforts are undertaken to increase students’ reflexivity. The same holds true for the postgraduate training during which all efforts are put on the delivery of knowledge and acquisition of skills, which provides the physician with the necessary means to applicate diagnostic and therapeutic procedures, but does not enable him to think about medicine and himself. Progress has been made with regard to the psychological and relational aspects in medicine, with the introduction of undergraduate and postgraduate communication training, medical psychology [55, 56] and different forms of group supervision such as groups aiming to support professional identify formation in medical residents, or focused on the clinician-patient relationship [67, 68]. However, these initiatives are still scarce and occupy only a small place in the under- and post-graduate learning catalogues. In addition, contextual factors shaping physicians’ lived experience are not always systematically addressed in these more psychologically oriented approaches. Reflexivity training may be an answer to fill in this gap [69]. While reflexivity and reflexivity training is still in an embryonic stage, its value, when conducted seriously and not in a superficial way, is without doubt an asset to situate and orient medical students and physicians in their complex, a demanding and evolving environment [70, 71]. Part of such trainings could be devoted to address the issue of calling, to elucidate its different facets and to situate it in the context of the study and practice of medicine.

The limitations of the study are linked to its design as an exploratory study, the fact that we did not use validated questionnaires, that the study has been conducted in a singular setting impeding the generalizability of results, and the existence of a possible selection bias, with non-participants of the survey being not interested in the subject, and who may have a quite different perspective on calling. In addition, social desirability despite the anonymous character of the survey or shame (with regard to not so noble motivators for studying medicine) may have diminished the mention or prevalence of certain responses regarding motivators to study medicine (money, family pressure, etc.).

## Conclusions

The findings of our study underline the relevance of calling as motivational force and reinforcer of study choice. Calling thus merits academic attention, be it as an object of scientific investigation or of teaching. Addressing calling in medical school as part of the reflexive part of the curriculum should take into account its non-utilitarian nature and its problematic effects when experienced as a requirement to be fulfilled. Calling has to be situated and critically approached by taking into account the learning context in which it operates.

## Data Availability

The datasets used and/or analyzed during the current study are available from the corresponding author on reasonable request.

## Abbreviations

ANOVA: Analysis of variance
B: Bachelor
FET: Fisher’s Exact Test
M: Master

## Footnotes

1 The hidden curriculum can be described as the “‘understandings,’ customs, rituals, and taken-for-granted aspects of what goes on in the life-space we call medical education” [15, 16]
